# Effects of a Personalized VLCKD on Body Composition and Resting Energy Expenditure in the Reversal of Diabetes to Prevent Complications

**DOI:** 10.3390/nu11071526

**Published:** 2019-07-04

**Authors:** Lorenzo Romano, Marco Marchetti, Paola Gualtieri, Laura Di Renzo, Meriann Belcastro, Gemma Lou De Santis, Marco Alfonso Perrone, Antonino De Lorenzo

**Affiliations:** 1 School of Specialization in Food Sciences, University of Rome Tor Vergata, 00133 Rome, Italy; 2 Section of Clinical Nutrition and Nutrigenomic, Department of Biomedicine and Prevention, University of Rome Tor Vergata, Via Montpellier 1, 00133 Rome, Italy; 3 Sadel, Casa di cura San Giuseppe, 88836 Cotronei (KR), Italy; 4 Division of Cardiology, University of Rome Tor Vergata, 00133 Rome, Italy

**Keywords:** diabetes, reversibility, obesity, nutrition, prevention, body composition, indirect calorimetry, lean mass, resting energy expenditure, VLCKD

## Abstract

The reversion of diabetes and the treatment of long-term obesity are difficult challenges. The failure mechanisms of rapid weight loss are mainly related to the wasting of lean mass. This single-arm study aims to evaluate the effects of a very low-calorie ketogenic diet (VLCKD) on body composition and resting energy expenditure in the short term reversal of diabetes mellitus Type 2. For eight weeks, subjects were administered a personalized VLCKD with protein intake based on lean mass and synthetic amino acidic protein supplementation. Each subject was assessed by anthropometry, Dual-energy X-ray Absorptiometry(DXA), bioimpedentiometric analysis (BIA), indirect calorimetry, and biochemical analysis. The main findings were the saving of lean mass, the reduction of abdominal fat mass, restored metabolic flexibility, the maintenance of resting energy expenditure, and the reversion of diabetes. These results highlight how the application of preventive, predictive, personalized, and participative medicine to nutrition may be promising for the prevention of diabetes and enhancement of obesity treatment.

## 1. Introduction

Obesity and poor nutrition are the main causes of the increase in chronic degenerative diseases that can be observed globally [1]. A recent publication showed that one fifth of deaths are due to a sub-optimal diet and ultra-processed food [2]. One of the main diseases related to obesity is diabetes mellitus type 2 (DMT2). The International Diabetes Federation reports that 382 million people worldwide suffer from DMT2 and that 352 million are at risk of developing it [3].

Limiting the incidence of diabetes and reducing the prevalence of subjects suffering from prediabetes and obesity would lead to a considerable improvement in the quality of life of patients and an economic savings governments. Paradoxically, although obesity is a cause of diabetes, the prescription of DMT2 drugs is significantly higher than those for anti-obesity, when it would be enough to treat obesity to reduce DMT2 onset [4]. Even more paradoxical is that personalized dietary interventions and lifestyle changes would be enough to cure obesity [5,6].

From the observation that the diabetic condition of patients undergoing bariatric surgery was improved, a twin cycle in the pathogenesis of DMT2 was hypothesized. According to the hypothesis of Taylor et al., the excess of calories was responsible for liver and pancreas lipotoxicity, insulin resistance, and beta cell differentiation [7]. Following this hypothesis, and in agreement with Lim et al. and Steven et al., it has been shown that a very low calorie diet (VLCD) may be effective in the treatment of obesity and reversal of DMT2 [8,9]. The ineffectiveness of long-term VLCDs is a common assumption and it is due to the loss of lean mass (LM) [10,11,12]. LM contributes to metabolism, performance, appetite, and finally to the daily calorie intake [13]. Furthermore, it was shown that at the end of a dietary treatment, a depletion of LM was responsible for exceeding the initial weight and collateral fattening [14]. Being vital, these phenomena are due to the organism’s need to restore the lean portion depleted during the restriction phase [15]. In addition, calorie restriction alone leads to poor compliance. One of the causes is the sense of hunger [10,11,12], a problem that is solved thanks to the anorexic effect of a ketogenic diet [16]. Currently, guidelines recommend the prescription of protein needs, in healthy or sick patients, according to the actual or ideal weight [17]. However, these recommendations do not consider the enormous variability of body composition (BC) present among individuals, especially for LM, which determines protein requirements [18,19]. In this regard, Geisler et al. concluded that the protein intake recommendations calculated on body weight are inaccurate. Moreover, they state that the prescription according to the LM is a necessity to avoid an overestimation or underestimation of the protein load [20]. Consequently, in the present study, a very low calorie ketogenic diet (VLCKD) with a protein intake based on LM, as per Colica et al. [21,22], and a synthetic amino acid powder supplementation as the only source of protein was prescribed. Previously, Merra et al. showed that the use of this supplement in a VLCKD resulted in a savings of appendicular muscle mass, decrease in visceral fat, and improvement of cardiovascular indices [23,24].

Finally, the aim of this single-arm study was to explore the effects of a VLCKD with synthetic amino acid protein supplementation on BC and resting energy expenditure (REE) in the short term reversal of DMT2. We hypothesized that with a personalized prescription of proteins in a VLCKD it would be possible to obtain a savings of LM, a maintenance of REE, and a high compliance. The results obtained will provide the precautions to be followed in the future to treat obesity and DMT2 in the long term more successfully and prevent dramatic complications with nutritional intervention alone.

## 2. Materials and Methods

### 2.1. Study Design

This prospective, single-center longitudinal single-arm study concerned a VLCKD for eight weeks in patients with DMT2. Biochemical-clinical analyses at baseline and at the end of the treatment were performed. Instrumental evaluations were performed at baseline, at four weeks of treatment, and at the end of the eight weeks. Rather than a comparative clinical study of treatment, this study aimed only to evaluate changes in DMT2 parameters, body composition, and basal metabolism following a VLCKD.

### 2.2. Subjects

At the Clinical Nutrition and Nutrigenomics section of the University of Rome “Tor Vergata”, 25 patients with DMT2 were enrolled. Inclusion criteria were as follows: age 25–75 years; DMT2 treated with diet and/or oral antidiabetics, dipeptidyl peptidase-4 inhibitors and/or insulin; and body mass index (BMI) between 18–45 kg/m^2^. Exclusion criteria were as follows: duration of illness over 8 years; weight loss over 5 kg in the previous 6 months; treatment with thiazolidinediones, glucagon-like peptide-1 agonists, steroids, or atypical antipsychotic; untreated thyroidism; renal alteration (serum creatinine > 1.7 mg/dL or 150 µmol/L) and habitual alcohol consumption. To exclude diagnostic errors, the diagnosis and evaluation of illness duration were performed by a specialist in diabetology, and this was confirmed by medical records. In addition to the dietary treatment, enrolled patients were asked not to change lipid-lowering therapy and lifestyle, while dosages for hypertension drugs were changed or reduced according to clinical needs throughout the study. With reference to diabetes therapy, enrolled patients were asked to interrupt all medications before the beginning of the VLCKD, as per Steven et al. [25]. All the enrolled subjects signed an informed consent, and the study protocol was approved by the ethical committee of the Calabria Region Center Area Section (Register Protocol No. 146 17/05/2018). Of the 25 patients enrolled, four were excluded according to the exclusion criteria and one did not start dietary treatment due to complications. Finally, 20 patients were included in the study, 10 women and 10 men.

### 2.3. Anthropometry

Body height and weight were assessed by a stadiometer and scale (SECA instruments, UK) while the patient was standing and only wearing underwear Data were collected to the nearest 0.1 cm and 0.1 kg, respectively. BMI was calculated as follows:

$$\mathrm{BMI}\;=\;\mathrm{body}\;\mathrm{weight}\;(\mathrm{kg})∕{\mathrm{height}}^{2}{\left(m\right)}^{2}$$

As done previously, four circumferences were measured: neck, abdomen, waist, and hip [26].

### 2.4. Body Composition and Resting Energy Expenditure

Body composition evaluation was performed after a 12 h overnight fast. Subjects underwent Dual-energy X-ray Absorptiometry (DXA) (Lunar i-DXA, GE Medical Systems, Milwaukee, WI, USA), with a specific system software package (EnCORE Software GE Healthcare, Chicago, IL, USA), to measure fat mass (FM) kg and %, both segmental and whole; LM kg and %; both segmental and whole; and bone mass (BM) kg. Before each testing session, standard DXA quality control and calibration measures were carried out.

Furthermore, each subject underwent a bioimpedentiometric analysis (BIA 101S, Akern/RJL Systems, Florence, Italy) to measure phase angle; resistance (Rz); reactance (Xc); total body water (TBW), L; extracellular body water (ECW), L; and body cell mass (BCM) kg [27,28].

An electronic dynamometer for measuring maximum handgrip strength was used (DynEx, Akern, Florence, Italy) [23].

Indirect calorimetry was performed to obtain REE, respiratory quotient (RQ), O_2_ and CO_2_ volumes (VO_2_ and VCO_2_), using a Vyntus CPX Canopy (CareFusion, Höchberg, Germany) with SentrySuite^TM^ Software (CareFusion, Höchberg, Germany). A gas mixture with 12.0% O_2_, 5.0% CO_2_, balanced with N_2_ was used. After a steady-state condition, when no variation over ± 5% occurred, VO_2_ and VCO_2_ values were recorded. According to the Weir equation, the REE was calculated without using urinary urea nitrogen levels. [29].

### 2.5. Analytical Procedures

Glycemia, insulinemia, HbA1c, hepatic transaminases, and creatinine at baseline and at eight weeks, after 12 hours of night fasting, were performed. The Homa Index was calculated to assess insulin resistance [22]. The analyses were performed at a Clinical Pathology Accredited laboratory.

### 2.6. Experimental Protocol

The included subjects undertook a dietary treatment: VLCKD. This treatment provided a synthetic amino acid powder supplementation of 8 grams per bag as the source of protein; it contained whey protein (6.71 g/bag), carbohydrates (0.015 g/bag), fats (0.075 g/bag), isoleucine (0.155 g/bag), ornithine alpha-ketoglutarate (0.125 g/bag), L-citrulline (0.125 g/bag), taurine (0.125 g/bag), L-arginine (0.100 g/bag), L-tryptophan (0.0375 g/bag), potassium citrate (0.100 g/bag), and pantothenic acid (0.0015 g/bag) for a total of 29 kcal (122 kJ) (Macresces, Italfarmacia s.r.l., Rome, Italy). The powder was dissolved in water and taken in daily meals. The amount of synthetic amino acid powder supplementation administered to each patient was calculated considering a supply of 2 g of proteins per kg of whole lean mass, measured by DXA, at baseline and after four weeks [25].

In addition, a quantity of vegetables equal to 600 g/day was administered, exclusively from non-starchy cooked and raw vegetables, subdivided into 2 or 3 portions during the day and a quantity of 20 mL of extra virgin olive oil per day, preferably raw. Finally, a minimum water intake of 2 L of mineral water per day was indicated. The average caloric content of the VLCKD was between 450–600 kcal/day for women and 650–800 kcal/day for men.

In summary, the average daily distribution of macronutrients and micronutrients was as follows: 5–10% of carbohydrates (<25 g/day), derived mainly from vegetables; 60–70% of proteins, mainly from the protein supplement and minimally from vegetables; 25–30% of lipids, exclusively from extra-virgin olive oil of which polyunsaturated fatty acids (PUFA) was <10 %; Monounsaturated Fatty Acids (MUFA) was 10–20 %, saturated fat was <5 %; and sodium < 2000 mg/day. All patients were informed about the VLCKD protocol and were asked to maintain their daily physical activity levels. To improve compliance, operators contacted patients during the treatment on a weekly basis.

### 2.7. Statistics

From the analysis of the literature of a previous work [30], it was observed that in the intervention group the mean difference between HbA1c (%) times was −0.9% and the calculated SD (∆) was 0.7. Therefore, to calculate the sample size for a before-and-after study, it was necessary to enroll 15 subjects, with a significance level of 5% and a study power of 80%. Taking into account a possible 20% follow-up loss, at 18 subjects should be enrolled. The data presented are expressed as mean, standard deviation, and as ∆%, to evaluate differences between the times. The two-tailed Student’s paired *t*-test or Wilcoxon rank test (if nonparametric) were used to assess the presence or not of differences in the variables examined between the established times. For each study variable, in order to compare the trend over time, ∆% were calculated equal to the percentage variation of each parameter calculated as an absolute margin of variation from the base value. The analyses were performed using the SPSS software (version 23, IBM, Armonk, New York, USA). All changes with *p* < 0.05 were considered significant.

## 3. Results

A total of 20 patients met the inclusion and exclusion criteria, 10 men and 10 women. Table 1 shows the characteristics of the study population and the antidiabetic drugs taken before starting the VLCKD. The age of the study participants was 56 ± 9.72 years. At baseline, subjects’ weights were 104.43 ± 18.85 kg and BMIs were 37.09 ± 6.83 kg/m^2^. Fasting glycemia were 170.06 ± 38.18 mg/dL and glycated hemoglobin were 7.33 ± 1.13% (57.06 ± 13.00 mmol/L) (Table 2, baseline column). 

### 3.1. Anthropometry

**Figure 1:**
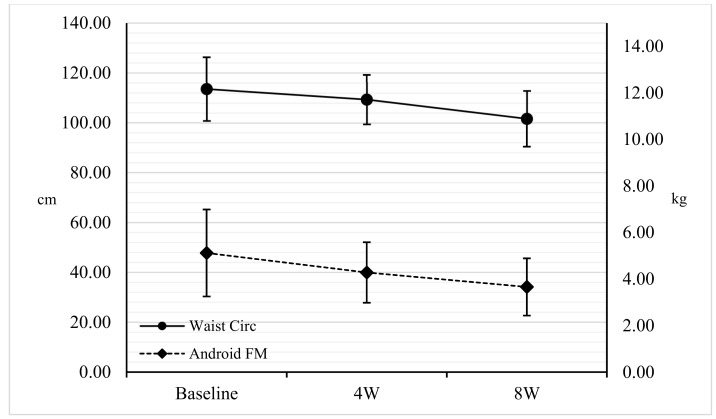
Comparison among baseline, four weeks, and eight weeks for waist circumference (cm) and android fat mass (kg). Points sharing the same superscript letter are not significantly different from each other. Statistical significance attributed to results with *p* < 0.05. Circ: Circumference. FM: Fat Mass; 4W: four weeks; 8W: eight weeks.

During the eight weeks of treatment, weight, BMI, and waist, abdomen, and hip circumferences were significantly reduced (*p* < 0.001). The neck circumference was significantly reduced (*p* < 0.001) in the first four weeks of treatment. The maximum force of the dominant hand did not show significant changes over time (Table 2 and Figure 1).

### 3.2. DXA

**Figure 2:**
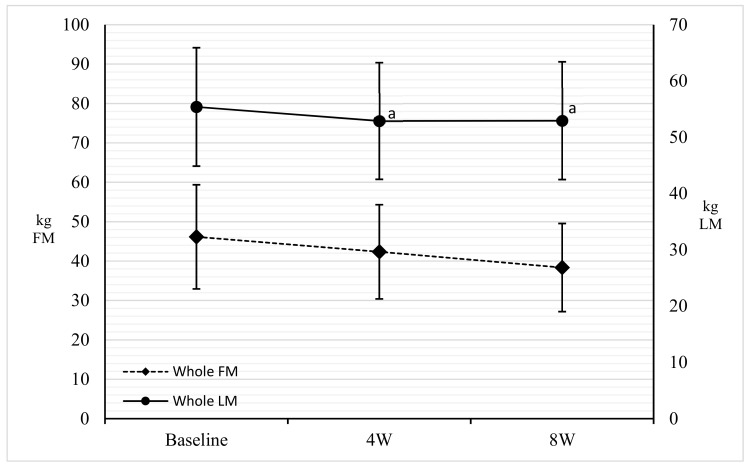
Comparison among baseline, four weeks, and eight weeks for whole FM and whole LM. Points sharing the same superscript letter are not significantly different from each other. Statistical significance attributed to results with *p* < 0.05. FM: Fat Mass; LM: Lean Mass; ECW: Extra Cellular Water; BCM: Body Cell Mass; 4W: 4 weeks; 8W: 8 weeks.

FM (kg), segmental and whole, showed a significant reduction (*p* < 0.001) in the three comparisons, except Arm FM, which was significantly reduced only between baseline and eight weeks (*p* = 0.01). Furthermore, a significant reduction (*p* < 0.001) of FM%, segmental and whole, with the exception of Leg and Gynoid FM%, which were significant between four weeks and eight weeks (*p* < 0.001), was observed (Figure 1). A significant reduction (*p* < 0.001) of the lean mass (kg), segmental and whole, was shown only between baseline and four weeks, while no variations were shown for the arms’ lean mass (kg) (Figure 2). Finally, BM (kg) showed no significant changes (Table 2).

### 3.3. BIA

**Figure 3:**
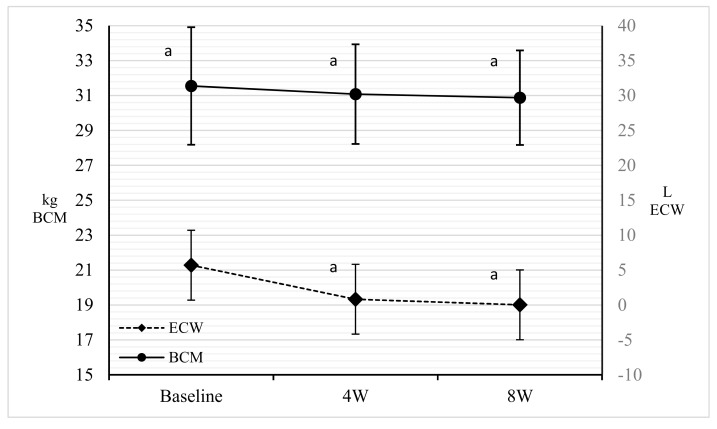
Comparison among baseline, four weeks, and eight weeks for ECW (L) and BCM (kg). Points sharing the same superscript letter are not significantly different from each other. Statistical significance attributed to results with *p* < 0.05. ECW: Extra Cellular Water; BCM: Body Cell Mass; 4W: 4 weeks; 8W: 8 weeks.

Both the Rz and the Xc underwent a significant increase (*p* < 0.001) between baseline and four weeks. Furthermore, TBW and ECW were significantly reduced (*p* < 0.001) between baseline and four weeks. Furthermore, no significant changes for BCM and phase angle (PA) were noted (Table 2 and Figure 2 and Figure 3).

### 3.4. Indirect Calorimetry

**Figure 4:**
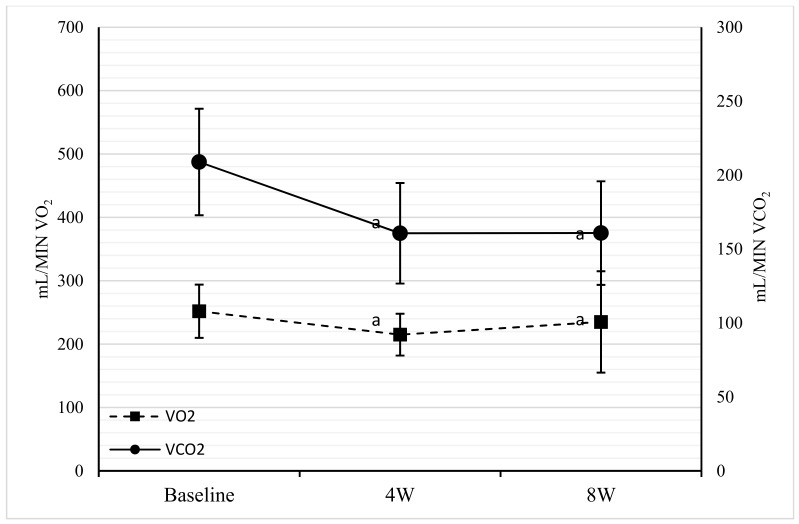
Comparison among baseline, four weeks, and eight weeks for VO_2_ and VCO_2_. Points sharing the same superscript letter are not significantly different from each other. Statistical significance attributed to results with *p* < 0.05. VO_2_: Volume of Oxygen; CO_2_: Volume of Carbon Dioxide; mL/MIN: milliliter/minutes; 4W: weeks; 8W: weeks.

Between baseline and four weeks, VO_2_, VCO_2_, RQ, and REE values were significantly reduced (respectively, *p* = 0.04; *p* = 0.04; *p* < 0.001; and *p* = 0.01), while no significant changes were observed between four weeks and eight weeks. Overall, a significant variation (*p* < 0.001) between baseline and eight weeks for VCO_2_, RQ, and REE was observed. Finally, no variation was noted for VO_2_ between four weeks and eight weeks, nor between baseline and eight weeks (Table 2 and Figure 4).

### 3.5. Blood Tests

All blood values evaluated between baseline and eight weeks were significantly reduced, except for creatinine, as shown in Table 3.

## 4. Discussion

In the process of DMT2 reversion, the main results obtained from this exploratory study on BC and REE changes are represented by the predominant reduction of abdominal fat mass, the saving of lean mass, and the reduction of fasting glycaemia and glycated hemoglobin. An acute weight loss occurred uniformly in the 20 patients who underwent eight weeks of VLCKD. Despite the rigidity of the nutritional treatment, we recorded 100% compliance. If personalized on individual parameters, this supports the hypothesis that a ketogenic diet is compliant and that it can induce a sense of satiety [16,31,32,33].

Weight loss at four weeks was −11.07% and at the end of the study, −15.77%. Waist, abdomen, and hips circumferences decreased during the eight weeks. These results can be linked to a reduction in cardiovascular and metabolic risk [34], normally very high in people with DMT2 [35,36]. Both waist and abdominal circumference had a 10% decrease. This reduction is reflected in the loss of FM% and FM (kg), equal to −17.75% and −8.10%, respectively [37,38]. In detail, it was shown that the loss affected the truncal fat by −20.72%, and above all the abdominal fat by −24.80%. This highlights that this type of nutritional treatment is useful in reducing the abdominal district fat mass, which is a site of accumulation of visceral fat [5,23]. A higher value of the latter corresponds to a greater accumulation of ectopic fat (hepato-pancreosteatosis) due to the spillover from saturated subcutaneous deposits, a process underlying the twin cycle [5,7].

An interesting fact was obtained by observing the variation over time. At the abdomen and trunk regions, typical sites of accumulation of android fat, the loss of FM in the first four weeks was significant. Only in the last four weeks of treatment was there a greater loss, even in appendicular fat. Similarly, the same phenomenon was observed by Gomez-Arbelaez et al. and indicates how the organism is able to respond to a caloric restriction with first a reduction in fat related to cardiometabolic risk and only subsequently of subcutaneous fat [39].

Compared to Taylor et al. who evaluated only weight loss without assessing BC, this study aimed to improve the concept of Personal Fat Threshold (PFT). Indeed, this element, defined by Taylor, could correspond to the FM% evaluated by DXA and recognized as a parameter for the diagnosis of obesity [26,40]. The main goal of a diet therapy aiming at the reversion of DMT2 is to obtain a normal value of FM% and not of BMI, which misclassifies body composition [41]. Together with the reversion of diabetes, it is hypothesized that the reduction in FM% and above all in abdominal fat observed in this study corresponds to the decrease in lipotoxicity below a hypothetical individual tolerance.

Regarding the saving of the LM, an initial loss was still observed between baseline and four weeks, but it stabilized by eight weeks. Despite this initial loss, our hypothesis on LM saving is supported by the maintenance of BCM and a significant decrease of ECW, thanks to a personalized protein prescription. The reduction of the water load is a known phenomenon in a low-calorie and ketogenic diet. In our study this is even more observable given the reduced sodium intake. The reduction in water content is due to the increase in diuresis due to urinary excretion of ketones, sodium, and the depletion of glucose reserves [42,43]. The saving of LM is both a milestone for therapeutic success [44] and an indicator of quality of the diet therapy, to avoid the relapsing effect due to the suspension of the hypocaloric treatment [5,45]. Our study confirms the results of Gomez-Arbeleaz et al. about the possibility of saving lean tissue during strongly low-calorie diets, even in diabetic patients [39]. According to different guidelines on obesity treatment, rapid weight loss with low-calorie diets is a cause of loss in muscle mass and strength [46]. On the contrary, to further support our hypothesis, the maintenance of maximum force was observed, as already demonstrated by other authors [23,39]. Finally, this fact refutes the assumption that a strongly hypocaloric diet induces loss of trophism and muscular efficiency [47].

From the metabolic point of view, a reduction of RQ (−12%) was observed corresponding to a change in the oxidized energy substrate, which moved towards fat, with a consequent increase in circulating fatty acids, as demonstrated by Hall et al. [44]. All patients treated with VLCKD changed from a condition of metabolic inflexibility to one of flexibility. This is a fundamental step in weight loss and in the reversibility of DMT2 due to the depletion of liver and pancreatic fat [7,48]. In addition to the restoration of metabolic flexibility, another goal was the association between maintaining the REE and saving the LM. It is generally accepted that low-calorie diets, accompanied by severe weight loss, cause a reduction in REE [10,11,12]. Leibel et al. [49] showed that energy expenditure in weight loss was mainly related to the metabolically active mass. With a 10% loss of weight, the REE dropped by ∼300 Kcal/day (14.5%) and with a 20% loss, the REE was reduced by ∼400 Kcal/day (20%). Instead, our results show a reduction in REE (<300 Kcal/day) only between baseline and four weeks, with an 11% weight loss. Also, a decrease in VO_2_ and an even bigger decrease in VCO_2_ with the restoration of the metabolic flexibility was observed. After four weeks and a weight loss >15% the REE and the gas volumes were not reduced further. Thanks to the BC assessment, we can state that the additional weight loss between four weeks and eight weeks was only due to the reduction in FM. Therefore, saving the LM also maintained the REE in the VLCKD. Metabolic adaptation can be attributed to the reduction of FM and related inflammatory mediators such as adipokines, and to the change of oxidized substrates. In addition, changes in leptin levels, thyroid hormones, sympathetic stimuli, and others not evaluated in this study may also be contributers [50].

Regarding the blood tests, this study led to a reduction in fasting glycemia of 39.7% and of glycated hemoglobin below 6.5%, corresponding to the return of subjects to within the normal ranges. These data confirm the results previously obtained in the Counterpoint [8] and Counterbalance [9] studies, even though some patients were treated with insulin for more than six years. Results similar to ours were observed in the eight-week clinical trial of Lim et al. [8,51], where a reduction in mean blood glucose from 166 mg/dL to 106 mg/dL and HbA1c from 7.4% to 6.0% was highlighted. Based on the results, short-term remission of diabetes has been achieved, but the long-term effects with the re-introduction of carbohydrates has not been explored.

Moreover, as already demonstrated by the Counterpoint study [8], a significant reduction in alanine aminotransferase (ALT) and insulin resistance was observed, supporting the reduction of hepatic and muscle insulin resistance. Our intervention exploits the hypothesized and demonstrated mechanisms by Taylor and Lim [7,8] and adds the assessment of changes in BC and REE parameters.

Finally, our single-center longitudinal single-arm study reinforced the hypothesis of the reversibility of diabetes and the twin cycle, adding the evaluation and saving of LM as fundamental in the process of reversion and therapeutic success. In the DiRECT study, Lean et al. achieved a one-year reversal of diabetes in 46% of treated patients and the maintenance of 15% weight loss in 24% of the treated [52]. At year two, the DiRECT program achieved the remission of one third of patients, demonstrating also that the maintenance of weight loss leads to remission [30]. As already observed similarly by Giordani et al., there was a maintenance of renal function with the caloric restriction and supplementation of amino acids used in our study [53]. In our study, no patients showed the adverse effects related to the intake of supplemented synthetic amino acids, such as nausea, dissociation, vertigo associated with cysteine, increases in urinary zinc excretion, headache, fatigue, nausea, and anorexia associated with histidine intake, and nausea, vomiting, and liver side effects due to methionine [54,55].

Therefore, we hypothesize that saving the LM and maintaining the REE will allow us to obtain long-term success, especially after a treatment that attacks visceral fat. However, the study has numerous limitations represented by the number of patients, the duration of the study, and the lack of follow-ups. Another limitation is the absence of a control arm, and for this reason the current results must be considered with caution. In the future, longitudinal and randomized case-control studies will be performed to confirm the results obtained in this first exploratory study. These results highlight how the application of preventive, predictive, personalized, and participative medicine to nutrition may be promising to prevent diabetes and enhance obesity treatment.

## Figures and Tables

**Table 1:** Characteristics and pharmacological treatment at baseline.

Subjects	20
Men	10
Women	10
Age (years)	56.13 ± 9.27
Diabetes Duration (years)	5.85 ± 1.73
Diabetes Treatment	
Diet (n)	8
Metformin (n)	15
Sulphonylurea (n)	5
Insulin (n)	10
Anti-Hypertensives (n)	15
Statins (n)	18

Data are expressed as mean ± standard deviation.

**Table 2:** Parameter changes during dietetic treatment (baseline, four weeks, eight weeks).

Parameters	Basal	Four Weeks	Eight Weeks	Δ Base–Four Weeks	Δ Four Weeks–Eight Weeks	Δ Base–Eight Weeks	*p* Base–Four Weeks	*p* Four Weeks–Eight Weeks	*p* Base–Eight Weeks
	Mean ± SD	Mean ± SD	Mean ± SD						
ANTHROPOMETRY									
Weight (kg)	104.43 ± 18.85	92.85 ± 27.61	89.07 ± 26.17	−11.07	−4.04	−15.77	0.000 *	0.000 *	0.000 *
BMI (kg/m^2^)	37.09 ± 6.83	34.75 ± 6.50	33.25 ± 5.99	−6.42	−4.12	−10.27	0.000 *	0.000 *	0.000 *
Neck circumference (cm)	43.08 ± 3.36	41.59 ± 3.22	40.92 ± 3.0’	−3.99	−1.35	−4.92	0.000 *	0.070	0.000 *
Waist circumference (cm)	113.56± 12.71	109.33 ± 9.86	101.65 ± 11.23	−4.87	−5.98	−10.37	0.000 *	0.000 *	0.000 *
Abdomen circumference (cm)	123.79 ± 12.96	119.21 ± 13.14	110.99 ± 12.52	−4.82	−6.12	−10.31	0.000 *	0.000 *	0.000 *
Hip circumference (cm)	118.65 ± 14.5	114.09 ± 12.58	108.32 ± 11.57	−3.38	−5.15	−8.38	0.000 *	0.000 *	0.000 *
Handgrip (dominant hand) (kg)	26.81 ± 8.03	27.34 ± 7.73	29.00 ± 7.32	1.60	2.23	3.39	0.640	0.070	0.050
DXA									
Arm FM (kg)	4.74 ± 1.30	4.36 ± 1.14	4.16 ± 1.17	−7.58	−7.39	−10.26	0.070	0.230	0.010 *
Leg FM (kg)	11.6 ± 4.18	10.97 ± 4.16	10.05 ± 3.75	−4.52	−8.21	−12.37	0.010 *	0.000 *	0.000 *
Trunk FM (kg)	28.35 ± 9.47	24.33 ± 6.68	21.33 ± 6.04	−9.64	−12.28	−20.72	0.000 *	0.000 *	0.000 *
Android FM (kg)	5.12 ± 1.87	4.28 ± 1.29	3.66 ± 1.23	−11.46	−14.96	−24.80	0.000 *	0.000 *	0.000 *
Gynoid FM (kg)	6.53 ± 2.05	5.97 ± 1.92	5.34 ± 1.63	−6.86	−10.13	−16.33	0.000 *	0.000 *	0.000 *
Whole FM (kg)	46.15 ± 13.22	42.35 ± 11.96	38.04 ± 11.18	−8.20	−10.36	−17.75	0.000 *	0.000 *	0.000 *
Arm FM (%)	43.26 ± 9.9	42.55 ± 9.24	42.22 ± 9.11	−2.41	−3.74	−6.36	0.010 *	0.000 *	0.000 *
Leg FM (%)	36.39 ± 9.56	36.64 ± 10.22	35.94 ± 10.41	−0.34	−4.87	−4.98	0.980	0.000 *	0.000 *
Trunk FM (%)	49.92 ± 6.6	48.32 ± 6.68	46.3 ± 7.18	−2.52	−6.13	−8.31	0.000 *	0.000 *	0.000 *
Android FM (%)	53.15 ± 6.34	51.48 ± 6.66	49.29 ± 7.52	−2.57	−6.10	−8.22	0.010 *	0.000 *	0.000 *
Gynoid FM (%)	42.51 ± 8.69	42.27 ± 9.24	41.19 ± 9.09	−1.13	−5.33	−6.38	0.200	0.000 *	0.000 *
Whole FM (%)	43.87 ± 7.38	42.89 ± 7.42	40.67 ± 7.59	−2.29	−6.13	−8.10	0.000 *	0.000 *	0.000 *
Arm LM (kg)	5.96 ± 1.84	5.74 ± 1.49	5.74 ± 1.69	−5.77	−1.78	−1.71	0.130	0.780	0.260
Leg LM (kg)	18.95 ± 4.26	17.97 ± 4.22	17.79 ± 4.46	−5.17	−1.37	−6.53	0.000 *	0.120	0.000 *
Trunk LM (kg)	26.56 ± 4.81	24.85 ± 4.69	24.54 ± 4.90	−5.30	−0.54	−6.09	0.000 *	0.490	0.000 *
Android LM (kg)	4.28 ± 0.89	3.91 ± 0.83	3.84 ± 0.85	−6.76	−1.59	−8.36	0.000 *	0.340	0.050
Gynoid LM (kg)	8.37 ± 1.63	7.85 ± 1.63	7.72 ± 1.71	−5.70	−1.54	−7.28	0.000 *	0.110	0.000 *
Whole LM (kg)	55.39 ± 10.52	52.9 ± 10.37	52.94 ± 10.46	−4.58	0.25	−4.49	0.000 *	0.790	0.000 *
Bone Mass (kg)	2.78 ± 0.59	2.80 ± 0.58	2.77 ± 0.59	−0.45	−1.02	−1.45	0.240	0.050	0.060
BIA									
Rz (Ohm)	471.89 ± 69.35	516.71 ± 75.68	508.00 ± 62.24	8.88	−0.75	8.56	0.000 *	0.730	0.040
Xc (Ohm)	47.84 ± 13.45	52.94 ± 10.38	51.86 ± 7.44	15.51	0.57	14.89	0.010 *	0.690	0.110
TBW (kg)	44.98 ± 7.88	41.65 ± 7.43	41.08 ± 7.03	−5.71	−1.57	−6.43	0.000 *	0.280	0.000 *
ECW (kg)	21.28 ± 4.37	19.33 ± 3.22	19.01 ± 2.87	−8.05	−2.11	−7.99	0.000 *	0.110	0.010 *
BCM (kg)	31.37 ± 8.42	30.2 ± 7.14	29.69 ± 6.77	0.67	1.36	−0.11	0.900	0.600	0.570
PA (°)	5.81 ± 1.58	5.89 ± 1.06	5.89 ± 0.90	6.09	1.43	6.26	0.180	0.940	0.730
CALORIMETRY									
VO_2_ (mL/min)	251.88 ± 42.7	214.92 ± 33.56	235.03 ± 88.42	−14.85	0.56	−6.27	0.040 *	0.970	0.410
VCO_2_ (mL/min)	208.88 ± 36.52	160.67 ± 34.26	160.81 ± 35.50	−20.49	−2.08	−23.80	0.040 *	0.510	0.000 *
RQ	0.83 ± 0.03	0.73 ± 0.04	0.73 ± 0.04	−12.37	0.07	−12.01	0.000 *	1.000	0.000 *
REE (kcal)	1784.50 ± 313.18	1435.33 ± 223.71	1498.00 ± 316.65	−16.83	1.94	−16.59	0.010 *	0.810	0.000 *

BMI: Body Mass Index; DXA: Dual-energy X-ray Absorptiometry; FM: Fat Mass; LM: Lean Mass; BIA: Bioimpedentiometry; Rz: Resistance; Xc: Reactance; TBW: Total Body Water; ECW: Extra Cellular Water; BCM: Body Cell Mass; PA: Phase Angle; VO_2_: Volume of Oxygen; VCO_2_: Volume of Carbon Dioxide; RQ: Respiratory Quotient; REE: Resting Energy Expenditure. All values are presented as mean ± standard deviation. * *p* < 0.05.

**Table 3:** Parameter changes during dietetic treatment (baseline, 8 weeks).

Parameters	Basal	Eight Weeks	*p*	∆ Base–Eight Weeks
	Mean ± SD	Mean ± SD		
Glycemia (mg/dL)	170.06 ± 11.18	99.67 ± 9.4	0.000 *	−39.70
HbA1c (%)	7.33 ± 0.35	6.16 ± 0.07	0.000 *	−15.73
HbA1c (mmol/L)	57.06 ± 3.2	44.06 ± 2.08	0.000 *	−21.83
Insulin (uU/mL)	17.89 ± 4.71	8.66 ± 3.64	0.000 *	−51.54
Homa Index	7.47 ± 2.07	2.13 ± 0.88	0.000 *	−71.39
AST (U/L)	36.75 ± 5.06	21.21 ± 4.49	0.030 *	−29.37
ALT (U/L)	45.08 ± 6.97	24.07 ± 5.69	0.000 *	−41.09
Creatinine (mg/dl)	0.81 ± 0.16	0.77 ± 0.14	0.040	−4.50

All values are presented as mean ± standard deviation. * *p* < 0.05. HbA1c: Hemoglobin A1c; AST: aspartate transaminase; ALT: alanine aminotransferase; ∆ difference between 8 weeks and baseline values.

## References

[1] Mozaffarian D., Angell S.Y., Lang T., Rivera J.A. (2018). Role of government policy in nutrition-barriers to and opportunities for healthier eating. BMJ.

[2] GBD 2017 Diet Collaborators (2019). Health effects of dietary risks in 195 countries, 1990–2017: A systematic analysis for the Global Burden of Disease Study 2017. Lancet.

[3] What Is Diabetes. https://www.idf.org/aboutdiabetes/what-is-diabetes/facts-figures.html.

[4] Thomas C.E., Mauer E.A., Shukla A.P., Rathi S., Aronne L.J. (2016). Low adoption of weight loss medications: A comparison of prescribing patterns of antiobesity pharmacotherapies and SGLT2s. Obesity (Silver Spring).

[5] Avolio E., Gualtieri P., Romano L., Pecorella C., Ferraro S., Di Renzo L., De Lorenzo A. (2019). Obesity and body composition in man and woman: Associated diseases and new role of gut microbiota. Curr. Med. Chem..

[6] Lehtisalo J., Lindström J., Ngandu T., Kivipelto M., Ahtiluoto S., Ilanne-Parikka P., Keinänen-Kiukaanniemi S., Eriksson J.G., Uusitupa M., Tuomilehto J. (2016). Diabetes, glycaemia, and cognition-a secondary analysis of the Finnish Diabetes Prevention Study. Diabetes Metab. Res. Rev..

[7] Taylor R., Al-Mrabeh A., Sattar N. (2019). Understanding the mechanisms of reversal of type 2 diabetes. Lancet Diabetes Endocrinol..

[8] Lim E.L., Hollingsworth K.G., Aribisala B.S., Chen M.J., Mathers J.C., Taylor R. (2011). Reversal of type 2 diabetes: Normalisation of beta cell function in association with decreased pancreas and liver triacylglycerol. Diabetologia.

[9] Steven S., Hollingsworth K.G., Al-Mrabeh A., Avery L., Aribisala B., Caslake M., Taylor R. (2016). Very Low-Calorie Diet and 6 Months of Weight Stability in Type 2 Diabetes: Pathophysiological Changes in Responders and Nonresponders. Diabetes Care.

[10] McMurray R.G., Soares J., Caspersen C.J., McCurdy T. (2014). Examining variations of resting metabolic rate of adults: A public health perspective. Med. Sci. Sports Exerc..

[11] Müller M.J., Bosy-Westphal A., Kutzner D., Heller M. (2002). Metabolically active components of fat-free mass and resting energy expenditure in humans: Recent lessons from imaging technologies. Obes. Rev..

[12] Grattan B.J., Connolly-Schoonen J. (2012). Addressing weight loss recidivism: A clinical focus on metabolic rate and the psychological aspects of obesity. ISRN Obes..

[13] Dulloo A.G., Jacquet J., Miles-Chan J.L., Schutz Y. (2017). Passive and active roles of fat-free mass in the control of energy intake and body composition regulation. Eur. J. Clin. Nutr..

[14] Dulloo A.G., Jacquet J., Girardier L. (1997). Poststarvation hyperphagia and body fat overshooting in humans: A role for feedback signals from lean and fat tissues. Am. J. Clin. Nutr..

[15] Rondanelli M., Talluri J., Peroni G., Donelli C., Guerriero F., Ferrini K., Riggi E., Sauta E., Perna S., Guido D. (2018). Beyond Body Mass Index. Is the Body Cell Mass Index (BCMI) a useful prognostic factor to describe nutritional, inflammation and muscle mass status in hospitalized elderly? Body Cell Mass Index links in elderly. Clin. Nutr..

[16] Sumithran P., Prendergast L.A., Delbridge E., Purcell K., Shulkes A., Kriketos A., Proietto J. (2013). Ketosis and appetite-mediating nutrients and hormones after weight loss. Eur. J. Clin. Nutr..

[17] Trumbo P., Schlicker S., Yates A.A., Poos M. (2003). Food and Nutrition Board of the Institute of Medicine, The National Academies. Dietary reference intakes for energy, carbohydrate, fiber, fat, fatty acids, cholesterol, protein and amino acids. J. Am. Diet. Assoc..

[18] Houston D.K., Nicklas B.J., Ding J., Harris T.B., Tylavsky F.A., Newman A.B., Lee J.S., Sahyoun N.R., Visser M., Kritchevsky S.B. (2008). Dietary protein intake is associated with lean mass change in older, community-dwelling adults: The Health, Aging, and Body Composition (Health ABC) Study. Am. J. Clin. Nutr..

[19] Campbell W.W., Trappe T.A., Wolfe R.R., Evans W.J. (2001). The recommended dietary allowance for protein may not be adequate for older people to maintain skeletal muscle. J. Gerontol. A Biol. Sci. Med. Sci..

[20] Geisler C., Prado C.M., Müller M.J. (2016). Inadequacy of Body Weight-Based Recommendations for Individual Protein Intake-Lessons from Body Composition Analysis. Nutrients.

[21] Colica C., Avolio E., Bollero P., Costa de Miranda R., Ferraro S., Sinibaldi Salimei P., De Lorenzo A., Di Renzo L. (2017). Evidences of a New Psychobiotic Formulation on Body Composition and Anxiety. Mediat. Inflamm..

[22] Colica C., Merra G., Gasbarrini A., De Lorenzo A., Cioccoloni G., Gualtieri P., Perrone M.A., Bernardini S., Bernardo V., Di Renzo L. (2017). Efficacy and safety of very-low-calorie ketogenic diet: A double blind randomized crossover study. Eur. Rev. Med. Pharmacol. Sci..

[23] Merra G., Gratteri S., De Lorenzo A., Barrucco S., Perrone M.A., Avolio E., Bernardini S., Marchetti M., Di Renzo L. (2017). Effects of very-low-calorie diet on body composition, metabolic state, and genes expression: A randomized double-blind placebo-controlled trial. Eur. Rev. Med. Pharmacol. Sci..

[24] Merra G., Miranda R., Barrucco S., Gualtieri P., Mazza M., Moriconi E., Marchetti M., Chang T.F., De Lorenzo A., Di Renzo L. (2016). Very-low-calorie ketogenic diet with aminoacid supplement versus very low restricted-calorie diet for preserving muscle mass during weight loss: A pilot double-blind study. Eur. Rev. Med. Pharmacol. Sci..

[25] Steven S., Taylor R. (2015). Restoring normoglycaemia by use of a very low calorie diet in long- and short-duration Type 2 diabetes. Diabet. Med..

[26] De Lorenzo A., Siclari M., Gratteri S., Romano L., Gualtieri P., Marchetti M., Merra G., Colica C. (2019). Developing and cross-validation of new equations to estimate fat mass in Italian population. Eur. Rev. Med. Pharmacol. Sci..

[27] Costa de Miranda R., Di Lorenzo N., Andreoli A., Romano L., De Santis G.L., Gualtieri P., De Lorenzo A. (2019). Body composition and bone mineral density in Huntington’s disease. Nutrition.

[28] Colica C., Di Renzo L., Trombetta D., Smeriglio A., Bernardini S., Cioccoloni G., Costa de Miranda R., Gualtieri P., Sinibaldi Salimei P., De Lorenzo A. (2017). Antioxidant Effects of a Hydroxytyrosol-Based Pharmaceutical Formulation on Body Composition, Metabolic State, and Gene Expression: A Randomized Double-Blinded, Placebo-Controlled Crossover Trial. Oxid. Med. Cell. Longev..

[29] De Lorenzo A., Di Renzo L., Morini P., de Miranda R.C., Romano L., Colica C. (2018). New equations to estimate resting energy expenditure in obese adults from body composition. Acta Diabetol..

[30] Lean M.E., Leslie W.S., Barnes A.C., Brosnahan N., Thom G., McCombie L., Peters C., Zhyzhneuskaya S., Al-Mrabeh A., Hollingsworth K.G. (2019). Durability of a primary care-led weight-management intervention for remission of type 2 diabetes: 2-year results of the DiRECT open-label, cluster-randomised trial. Lancet Diabetes Endocrinol..

[31] Westerterp-Plantenga M.S., Nieuwenhuizen A., Tome D., Soenen S., Westerterp K.R. (2009). Dietary protein, weight loss, and weight maintenance. Annu. Rev. Nutr..

[32] Veldhorst M., Smeets A., Soenen S., Hochstenbach-Waelen A., Hursel R., Diepvens K., Lejeune M., Luscombe-Marsh N., Westerterp-Plantenga M. (2008). Protein-induced satiety: Effects and mechanisms of different proteins. Physiol. Behav..

[33] Paoli A., Cenci L., Fancelli M., Parmagnani A., Fratter A., Cucchi A., Bianco A. (2010). Ketogenic diet and phytoextracts. Comparison of the efficacy of mediterranean, zone and tisanoreica diet on some health risk factors. Agro Food Ind. Hi-Tech.

[34] Acconcia M.C., Caretta Q., Romeo F., Borzi M., Perrone M.A., Sergi D., Chiarotti F., Calabrese C.M., Sili Scavalli A., Gaudio C. (2018). Meta-analyses on intra-aortic balloon pump in cardiogenic shock complicating acute myocardial infarction may provide biased results. Eur. Rev. Med. Pharmacol. Sci..

[35] Arnett D.K., Blumenthal R.S., Albert M.A., Buroker A.B., Goldberger Z.D., Hahn E.J., Himmelfarb C.D., Khera A., Lloyd-Jones D., McEvoy J.W. (2019). 2019 ACC/AHA Guideline on the Primary Prevention of Cardiovascular Disease: A Report of the American College of Cardiology/American Heart Association Task Force on Clinical Practice Guidelines. J. Am. Coll. Cardiol..

[36] Guthrie N., Runyan J.W., Clark G., Marvin O. (1964). Carbohydrate Intake and Respiratory Quotient. Nutr. Rev..

[37] Steven S., Carey P.E., Small P.K., Taylor R. (2015). Reversal of Type 2 diabetes after bariatric surgery is determined by the degree of achieved weight loss in both short- and long-duration diabetes. Diabet. Med..

[38] Di Renzo L., Carbonelli M.G., Bianchi A., Iacopino L., Fiorito R., Di Daniele N., De Lorenzo A. (2012). Body composition changes after laparoscopic adjustable gastric banding: What is the role of -174G>C interleukin-6 promoter gene polymorphism in the therapeutic strategy?. Int. J. Obes. (Lond.).

[39] Gomez-Arbelaez D., Bellido D., Castro A.I., Ordoñez-Mayan L., Carreira J., Galban C., Martinez-Olmos M.A., Crujeiras A.B., Sajoux I., Casanueva F.F. (2017). Body Composition Changes After Very-Low-Calorie Ketogenic Diet in Obesity Evaluated by 3 Standardized Methods. J. Clin. Endocrinol. Metab..

[40] Overweight and Obesity. https://www.who.int/gho/ncd/risk_factors/overweight_obesity/obesity_adults/en/.

[41] De Lorenzo A., Romano L., Di Renzo L., Gualtieri P., Salimei C., Carrano E., Rampello T., de Miranda R.C. (2019). Triponderal mass index rather than body mass index: An indicator of high adiposity in Italian children and adolescents. Nutrition.

[42] Kolanowski J., Bodson A., Desmecht P., Bemelmans S., Stein F., Crabbe J. (1978). On the relationship between ketonuria and natriuresis during fasting and upon refeeding in obese patients. Eur. J. Clin. Investig..

[43] Frigolet M.E., Ramos Barragán V.E., Tamez González M. (2011). Low-carbohydrate diets: A matter of love or hate. Ann. Nutr. Metab..

[44] Hall K.D., Chen K.Y., Guo J., Lam Y.Y., Leibel R.L., Mayer L.E., Reitman M.L., Rosenbaum M., Smith S.R., Walsh B.T. (2016). Energy expenditure and body composition changes after an isocaloric ketogenic diet in overweight and obese men. Am. J. Clin. Nutr..

[45] Bray G.A., Kim K.K., Wilding J.P.H., World Obesity Federation (2017). Obesity: A chronic relapsing progressive disease process. A position statement of the World Obesity Federation. Obes. Rev..

[46] Pi-Sunyer F.X., Becker D.M., Bouchard C., Carleton R.A., Colditz G.A., Dietz W.H., Foreyt J.P., Garrison R.J., Grundy S.M., Hansen B.C. (1998). Clinical guidelines on the identification, evaluation, and treatment of overweight and obesity in adults: Executive summary. Expert Panel on the Identification, Evaluation, and Treatment of Overweight in Adults. Am. J. Clin. Nutr..

[47] National Institute for Health and Care Excellence Obesity: Identification, assessment and management of overweight and obesity in children, young people and adults. In NICE Clinical Guidelines; Puo, C., Ed.; 2014. https://www.nice.org.uk/guidance/cg189.

[48] Goodpaster B.H., Sparks L.M. (2017). Metabolic Flexibility in Health and Disease. Cell Metab..

[49] Leibel R.L., Rosenbaum M., Hirsch J. (1995). Changes in energy expenditure resulting from altered body weight. N. Engl. J. Med..

[50] Carrasco F., Papapietro K., Csendes A., Salazar G., Echenique C., Lisboa C., Díaz E., Rojas J. (2007). Changes in resting energy expenditure and body composition after weight loss following Roux-en-Y gastric bypass. Obes. Surg..

[51] Lim E.L., Hollingsworth K.G., Smith F.E., Thelwall P.E., Taylor R. (2011). Inhibition of lipolysis in Type 2 diabetes normalizes glucose disposal without change in muscle glycogen synthesis rates. Clin. Sci. (Lond.).

[52] Lean M.E., Leslie W.S., Barnes A.C., Brosnahan N., Thom G., McCombie L., Peters C., Zhyzhneuskaya S., Al-Mrabeh A., Hollingsworth K.G. (2018). Primary care-led weight management for remission of type 2 diabetes (DiRECT): An open-label, cluster-randomised trial. Lancet.

[53] Giordani I., Malandrucco I., Donno S., Picconi F., Di Giacinto P., Di Flaviani A., Chioma L., Frontoni S. (2014). Acute caloric restriction improves glomerular filtration rate in patients with morbid obesity and type 2 diabetes. Diabetes Metab..

[54] Garlick P.J. (2004). The nature of human hazards associated with excessive intake of amino acids. J. Nutr..

[55] Imamura W., Yoshimura R., Takai M., Yamamura J., Kanamoto R., Kato H. (2013). Adverse effects of excessive leucine intake depend on dietary protein intake: A transcriptomic analysis to identify useful biomarkers. J. Nutr. Sci. Vitaminol..

